# Supplementary material for: Intermittent catheter users’ symptom identification, description and management of urinary tract infection: a qualitative study
Source: BMJ Open. 2017 Sep 3;7(9):e016453. doi: 10.1136/bmjopen-2017-016453 (PMC5588948; doi:10.1136/bmjopen-2017-016453)
Supplement: Supplementary file 2 [file bmjopen-2017-016453supp002.pdf]

The MultICath Study (LREC: 13/LO/1511) - Urinary Tract Infection (UTI) signs and symptoms

Participant study ID: _______________ **Date of completion:** ________________ Completed by (interviewer name): ____________________

Tick all the signs and symptoms that refer to your experience of UTI:

**Signs and symptoms**													
	**Autonomic dysreflexia**	**Fever**	**Kidney /Bladder discomfort**	**Increased Bladder activity (spasticity)**	**Feeling sick**	**Sense of unease**	**Increased catheterising**	**Feeling tired**	**Incontinence**	**Foul smelling urine**	**Cloudy urine**	**White blood cells in urine**	**Haematuria**
Tick if yes													
**Circle how often you experience each symptom when you get a UTI**													
Always													
Sometimes													
Rarely													

Which of the above symptoms they mentioned would cause them to seek help from GP? ____________________________

Which of the above symptoms they mentioned would cause them to take antibiotics (patients with own supply)? ____________________________

How often does the patient have a UTI? (tick which applies)

**Once a week**	**Once a month**	**Every other month**	**Twice a year**	**Once a year**	**Less than once a year**

How many times has the patient been to their GP in the last year <u>for a UTI</u>? __________________________________

How many times has the patient taken antibiotics in the last year <u>for a UTI</u>?__________________________________

Have you been referred to a specialist in the last year for UTI symptoms? (eg. Urologist) ______________________
