# Supplementary material for: Intermittent catheter users’ symptom identification, description and management of urinary tract infection: a qualitative study
Source: BMJ Open. 2017 Sep 3;7(9):e016453. doi: 10.1136/bmjopen-2017-016453 (PMC5588948; doi:10.1136/bmjopen-2017-016453)
Supplement: Author's manuscript [file bmjopen-2017-016453.draft_revisions.pdf]

BMJ Open is committed to open peer review. As part of this commitment we make the peer review history of every article we publish publicly available.

When an article is published we post the peer reviewers' comments and the authors' responses online. We also post the versions of the paper that were used during peer review. These are the versions that the peer review comments apply to.

The versions of the paper that follow are the versions that were submitted during the peer review process. They are not the versions of record or the final published versions. They should not be cited or distributed as the published version of this manuscript.

BMJ Open is an open access journal and the full, final, typeset and author-corrected version of record of the manuscript is available on our site with no access controls, subscription charges or pay-per-view fees (http://bmjopen.bmj.com).

If you have any questions on BMJ Open's open peer review process please email info.bmjopen@bmj.com

BMJ Open

Intermittent catheter users' symptom identification, description, and management of urinary tract infection: a qualitative study

Journal:	*BMJ Open*
Manuscript ID	bmjopen-2017-016453
Article Type:	Research
Date Submitted by the Author:	16-Feb-2017
Complete List of Authors:	Okamoto, Ikumi; University of Southampton, Faculty of Health Sciences<br>Prieto, Jacqui; University of Southampton, Faculty of Health Sciences<br>Avery, Miriam; University of Southampton, Faculty of Health Sciences<br>Moore, Katherine ; University of Alberta<br>Fader, Mandy; University of Southampton, Faculty of Health Sciences
**Primary Subject Heading**:	Urology
Secondary Subject Heading:	Urology, Qualitative research
Keywords:	Urinary tract infections < UROLOGY, QUALITATIVE RESEARCH, PRIMARY CARE

SCHOLARONE™
Manuscripts

For peer review only

Intermittent catheter users' symptom identification, description, and management of urinary tract infection: a qualitative study

Corresponding author
Mandy Fader
Faculty of Health Sciences, University of Southampton, South Academic Block (MP11), Southampton General Hospital, Tremona Road, Southampton SO16 6YD
m.fader@soton.ac.uk
(023) 8059 6677

Authors
Ikumi Okamoto[1]
Jacqui Prieto[1]
Miriam R. Avery[1]
Katherine N. Moore[2]
Mandy Fader[1]

[1]Faculty of Health Sciences, University of Southampton, South Academic Block (MP11), Southampton General Hospital, Tremona Road, Southampton SO16 6YD
[2] Faculty of Nursing, University of Alberta, Edmonton, Canada

Word count

3923 words at the moment (from Intro to Conclusion excluding tables)

For peer review only

For peer review only - http://bmjopen.bmj.com/site/about/guidelines.xhtml

ABSTRACT

Objectives
To elucidate the views of intermittent catheter (IC) users regarding urinary tract infection (UTI) symptom presentation, terminology for describing signs and symptoms, the cause of UTI and management strategies.

Design
Qualitative study with semi-structured interviews. The transcribed text was analysed thematically.

Setting
12 GP surgeries in Hampshire and Dorset, UK.

Participants
A convenience sample of 30 IC users; aged over 18, using IC for at least 3 months who had at least one self-reported UTI since starting IC.

Results
Over 80% of participants reported urine cloudiness and smell as the indicators of UTI. The terms they used often differed from those in the NIDRR symptom set. Individuals had difficulty distinguishing possible UTI symptoms from those of their co-morbidities. They expressed uncertainty about the cause of UTI, often attributing it to poor hygiene and lifestyle behaviours. Whereas some viewed UTI as an expected consequence of IC use that could be self-managed, others felt more concerned and more reliant on their General Practitioner (GP). A range of coping strategies were described, including drinking more fluids, increased attention to personal hygiene and self-medicating with antibiotics.

Conclusions
The study shows that there is uncertainty among IC users about signs and symptoms of UTI and when to seek help. Individual descriptions of UTI fit generally within the NIDRR descriptors but may require more user friendly language. IC users' experience of UTI signs and symptoms can lack precision, owing partly to the presence of underlying health conditions. This, together with a need to accurately describe their signs and symptoms and the differing levels of concern about the need to seek help, presents challenges for the GP. This study provides the basis for developing a self-help tool which may aid identification of UTI and enhance communication with health care professionals.

Strengths and limitations of this study

- This is the first qualitative study to explore IC users' views, experiences, and descriptions of UTI signs and symptoms.
- The study includes IC users who had a wide range of reasons for using IC, including both neurological or non-neurological conditions.
- All UTI signs and symptoms discussed in this paper were subjective, retrospective, and self-reported, and were not confirmed by laboratory analysis.
- Advice from heatlh care professionals that IC users discussed was not confirmed.

For peer review only - http://bmjopen.bmj.com/site/about/guidelines.xhtml

INTRODUCTION

Intermittent catheterisation (IC) is widely used for the urological management of people with incomplete bladder emptying.[1 2] Urinary tract infection (UTI) is the most frequently reported and challenging complication.[2] In 1992 medical experts developed a key document on diagnosis and management of UTI in spinal cord injured individuals (SCI) for the National Institute on Disability and Rehabilitation Research (NIDRR).[3] The signs and symptoms listed in 1992 remain the current clinical standard to guide practice for the neurogenic patient: positive urine culture plus one or more of leukocytes in the urine, discomfort or pain over the kidney or bladder or during voiding, onset of urinary incontinence, fever, increased spasticity, autonomic dysreflexia, cloudy malodorous urine, or malaise, lethargy, sense of unease. However, the document did not include patient contributions to the terminology nor to the presenting symptoms. As UTI signs and symptoms can be difficult for some IC users to interpret, the NIDRR descriptors may be perceived differently or may not directly apply [2 4] and thus can be difficult for an individual to describe to their general practitioner (GP). In one study of UTI in people with SCI using IC, only 66% were able to self-predict a UTI when compared with NIDRR-accepted criteria.[5] Research exploring how IC users themselves identify and describe the signs and symptoms and comparing individual responses to the NIDRR criteria may aid in understanding how signs and symptoms are interpreted and when assistance is sought; patient oriented language may also assist in mutual understanding of the presence of a UTI.

Although there are studies exploring experiences and issues related to IC,[6-12] UTI has not been a focus. We found no qualitative research specifically exploring IC users' descriptions of UTI signs and symptoms nor how these descriptions compare to the NIDRR symptom list. Thus in this study, we conducted qualitative interviews with IC users with the aim of investigating their descriptions of UTI signs and symptoms, including their strategies for prevention, identification,

For peer review only - http://bmjopen.bmj.com/site/about/guidelines.xhtml

and management, with the goal of informing further research on a symptom-specific tool for IC users.

METHOD

The study used a qualitative method. Convenience sampling was used to recruit participants using IC on a regular basis from 12 GP practices in Hampshire and Dorset, UK. Participants were over 18 years of age, had independently used IC for at least 3 months and had no reported urethral deformities or immune deficiency disease.

Data collection

One face to face interview lasting 45-60 minutes was conducted in each participant's home by one of three experienced female interviewers including a qualitative researcher (SS, PhD) and research nurses (BC, BSc and MA, PhD).. None of the interviewers had had previous contact with any of the participants, and they attended as a non-clinical and non-judgemental listener. If desired, a relative or carer of participant was present. Appendix 1 shows the semi-structured interview guide. Using UTI signs and symptoms reported by the first six participants combined with the NIDRR symptom set[3] and the symptom set used in Massa et al[5] we derived a list (Appendix 2) to use as a prompt in subsequent interviews. Participants were first asked to describe their UTI signs and symptoms, and were then prompted with the symptom list to recollect any further details. Recruitment continued until data saturation was reached. Interviews were recorded and then transcribed verbatim. All transcripts were anonymised with an assigned study number. The transcripts were read only by the researchers and not by participants. Field notes were made during interviews, which were used in the analysis.

Data analysis

A qualitative researcher (IO, PhD) who had not conducted the interviews analysed the data thematically and coded it using NVivo 10 (QSR International). The first 20 transcripts formed an

For peer review only

For peer review only - http://bmjopen.bmj.com/site/about/guidelines.xhtml

initial coding framework. Coding was further refined through discussion with the wider research team and ongoing analysis of the remaining transcripts. Newly emerged themes that did not fit into the initial themes were discussed as a team. No participants were involved in the analysis of data.

RESULTS

Participants

One hundred and thirty-nine IC users were invited to take part and 74 (53%) responded of whom 42 were willing to participate. Of those, 3 participants were found ineligible. Furthermore, 9 participants were excluded from this study as they had never experienced UTI since starting IC. The total number of participants was therefore 30, including 19 men and 11 women, mean age 66 years (range 23-86), using IC for approximately 10 years (range 9 months – 31 years). The frequency of IC ranged from 1-10 cathetersiations a day (mean 4/day). Reasons for IC included chronic urinary retention (N=15), neurological impairment (N=10), and other factors such as post surgery or post chemotherapy (N=5). Three (10%) were currently re-using their catheters but as they were few in number, no distinction was made between re-users and single-users during data analysis.

Findings

Findings are presented in three main themes: 1) IC users' experiences of UTI and description of their signs and symptoms; 2) help-seeking actions when UTI is suspected ; and 3) UTI prevention and understanding the causes.

1 <u>Experiences of UTI and description of their signs and symptoms</u>

1.1 Signs and symptoms of perceived UTI

The percentage of participants citing each signs and symptoms is given in Table 1 and shows that the majority (80%) stated that odourous or cloudy urine were the most common indicators

For peer review only - http://bmjopen.bmj.com/site/about/guidelines.xhtml

of UTI, 50% also described changes in voiding function (frequency, burning), and 40% described general symptoms such as “feeling unwell”. Pain and discomfort were less often cited. Table 1 provides detailed descriptions of the various signs and symptoms attributed to UTI with a corresponding comparison to NIDRR clinical criteria.

Table 1: Signs and symptoms of UTI described and mapped to NIDRR consensus statement

	**Perceived signs and symptoms of UTI described by IC users**	**Mapping to the NIDRR consensus statement**	**N (%) Total=30**
Changes to urine	• Different colour<br>• Cloudiness<br>• Darkness in the urine<br>• A bit like rather thick soup	Cloudy urine	24 (80%)
	• The smell of urine not being right<br>• A really strong smell<br>• Smell<br>• Horrible smell<br>• Change in the odour of the urine	Urine with increased odour	22(73%)
	• Looked like there was a little bit of blood present<br>• Specks of blood		4 (13%)
	• There was protein present (found at GP)	Leukocytes in the urine generated by the mucosal lining	2 (7%)
Changes in bladder emptying	• Feel like you need to go more often but not passing enough<br>• I was going to the toilet, and then not feeling as I’d actually been; there was still a pressure there of wanting to go<br>• You feel you want to keep having a pee constantly<br>• Having to go more frequently<br>• Can’t pass water properly<br>• Be able to have a normal pee without having to use a catheter<br>• I feel as though I empty my bladder continually<br>• Going to toilet less		15 (50%)

For peer review only - http://bmjopen.bmj.com/site/about/guidelines.xhtml

	• Leak a bit between catheterisation<br>• Cannot hold my water; a couple of accidents during the day	Onset of urinary incontinence	4 (13%)
General symptoms	• Not feeling well<br>• I feel low, very rundown<br>• don't feel right<br>• lesser feeling of well-being; slight feeling of discomfort, not quite right<br>• don't feel comfortable	Malaise, lethargy	12 (40%)
	• Hot and sticky<br>• Bad fever, high temperature<br>• Very hot and shivery, not much of a temperature<br>• A fever symptom	fever	8 (27%)
	• I feel like I don't want to go anywhere and I feel tired<br>• In terms of worrying about needing to go to the loo a lot more<br>• I just worried about (UTI)<br>• Don't feel right in yourself	Sense of unease	4 (13%)
	• Vertigo<br>• Felt as if I was going to pass out<br>• Couldn't eat anything; constantly being sick; diarrhoea<br>• Devastating		4 (13%)
Pain/ discomfort	*Discomfort or pain over bladder*<br>• The bladder gets very irritated; the whole pelvis is quite irritated<br>• Burning bladder<br>• The pain usually starts in the urethra area<br>• Slight irritation (in my urethra and around that area)	Discomfort or pain over the kidney or bladder, or during urination	9 (30%)
	Discomfort or pain over kidney<br>• tremendous pain in my back, in my kidney area<br>• dull ache in my lower back and stinging		3 (10%)

For peer review only

	Pain during urination<br>• Get a pain when you are having a pee<br>• Burning when I pass urine<br>• Very difficult to pass water<br>• When I go to the loo, it slightly burns		5 (17%)
	Discomfort or pain in penis/groin area<br>• Stinging feeling in your penis itself		6(20%)
	Non-localised discomfort/pain<br>• Feel uncomfortable down below<br>• Get uncomfortable; when I sit down, feels like there is something there; uncomfortable to walk<br>• It's really really painful<br>• I wouldn't say painful, but it's discomfort<br>• Pain, stinging sometimes<br>• Feeling uncomfortable; even if I've used a catheter, still feels uncomfortable<br>• It burns like mad. There's no part that doesn't burn<br>• a tenderness all around the area<br>• everything down there feels very irritable; feel very uncomfortable<br>• it gets extremely painful		3 (10%)
	Not reported	Increased spasticity	
	Not reported	Autonomic dysreflexia	

1.2 Terms used to describe signs and symptoms

Participants were likely to use informal terms to describe their signs and symptoms such as 'not feeling right or 'feeling uncomfortable' rather than the NIDRR symptoms 'sense of unease', 'malaise' or 'lethargy'. If they described pain it was generalised and only localised if prompted.

For peer review only
For peer review only - http://bmjopen.bmj.com/site/about/guidelines.xhtml

Terms such as ‘discomfort’ and ‘uncomfortable’ were used to describe both general physical symptoms including pain or even increased urinary frequency – ‘*everything feels very irritable … in the area of the penis or the bladder. You just feel very uncomfortable …as if you want to go all the time’ (Particpant 41, male)*. Corresponding terms in the NIDRR set such as ‘kidney/bladder discomfort’ did not cover the breadth of symptoms experienced.

1.3 Uncertainty about whether symptoms were caused by UTI

Deciding if they had a UTI was difficult for some participants because of co-morbid conditions. For example, one with bladder cancer and “prostate problems” sometimes had a feeling of irritation but was unsure whether symptoms were caused by UTI or by his other health problems:

> *[Talking about UTI is] difficult because I’ve always had problems down below. I mean I [have] prostate problems, bladder problems, bladder cancer, a lot. So sometimes, I get this irritation feeling but [it] never bothers me. (Participant 12, male)*

Others also mentioned the difficulty of distinguishing UTI symptoms from their age-related problems.

> *I’m eighty-four and it’s one of those things, you expect things to happen as you’re getting old. Aches and pains, and stuff like that, it could be to do with that, I don’t know, but you get so many bits and pieces. (Participant 4, male)*

For peer review only - http://bmjopen.bmj.com/site/about/guidelines.xhtml

2 Help-seeking actions when UTI is suspected

2.1 Decision process whether to seek help or not

How participants managed perceived symptoms varied considerably. Most usually waited a few days to be convinced that their symptoms were caused by UTI before contacting their GP and used ‘self-help’ strategies such as increasing fluids:

> *[I] try to drink plenty and sort of flush it through and obviously if after a day or two I still felt bad or got worse I would have gone to the doctor's. (Participant 3, female)*

Others watched and waited until the onset of other symptoms, such as pain and discomfort, in addition to the first signs.

> *I think it's a little bit cloudy, but the next day it's perfectly clear. So I think one gets this in the way, sometimes, and if it is notably cloudy and I'm getting the pain [in the penis], it becomes pretty obvious that actually that's what I've got. (Participant 32, male)*

Another stated:

> *Since [I started IC] I have had and may well even now have a urinary infection, but if I drink enough it just doesn't affect me. Would I want to be on antibiotics all the time? […] I think it is more important that [UTI] is not affecting your overall health. I probably have an infection at the moment but it doesn't affect me in any way, I don't have a temperature, I'm not ill as a result of it. I actually had it out with my doctor a bit because they did a test at one point and said that I had an infection and I must take antibiotics. I thought I*

For peer review only - http://bmjopen.bmj.com/site/about/guidelines.xhtml

can but I'll probably have another one in 3 or 6 months time. (Participant 37, male)

Others contacted the GP at the first sign, such as cloudy/smelly urine, as previous experience suggested more severe symptoms would follow if left untreated:

I always know as soon as I've got [an infection] because as soon as it's there, the smell's there. And if I don't sort of act on it straight away … within a week I'll be feeling really unwell. […] And if I leave it later, it takes a lot longer to get rid of it. (Participant 11, female)

A participant's decision to consult their GP was also influenced by previous advice they had received about UTI. For example, a participant with cauda equina who '*lost most feeling below the waist*' said his GP had told him, *'You probably will know when you've got [UTI] if you get this sort of sickly-sweet smell.'(Pariticipant 10, male)*

2.2 Medication with antibiotics

If self-help strategies weren't effective, over 50% of individuals contacted the GP for a prescription for antibiotics. Three self-medicated with antibiotics which they kept at home. One felt that:

'If you keep on having antibiotics that eventually it's not going to work'. (Participant 13, male)

For another, a supply of antibiotics was important as it reassured him that he had ready access to treatment:

It's funny, but if I had an infection, it's usually on a weekend, I can't catch anybody to get the antibiotics or whatever I need, so then I'd have to go to the

For peer review only - http://bmjopen.bmj.com/site/about/guidelines.xhtml

hospital and all that palaver. So I just keep one in stock all the time. (Participant 19, male)

[…] Sometimes [infection] will start by a burning in my willy [penis], and then it will back up further to the bladder, [but] I don't have that problems anymore, because I immediately put a pill into my mouth and off I go and three days later it's gone. (Participant 19, male)

Seven took daily prophylactic antibiotics. One, using IC for 3 years, said:

I still get infections, off and on. You know when you get an infection, but I've taken tablets all the time for it. Every day, for infection. The doctor gave them to me, saying 'as soon as you're on a catheter, you've got to take these.' (Participant 4, male)

One participant used a urine dipstick test along with self administered antibiotics as suggested by the GP. She valued this approach and explained that it enabled her to act on UTI promptly and avoid a trip to the GP surgery and risk seeing a physician who did not know her history:

You've got all the signs for a water infection [smell of urine, feeling hot and sticky, and leaking of urine], by the time you've got that appointment, you could have had that water infection for five days so you know what my old GP did? He sort of said you can go on the internet and you can buy the sticks to test your water yourself which we did, so I've got the sticks myself and my GP once a year tends to give me a prescription a repeat prescription for a couple of courses of antibiotics. (Participant 6, female)

2.3 Relationship with GPs

Having a good relationship with and an easy access to the GP was greatly valued:

For peer review only

[My GP]'s obviously well aware of where I'm at and he looks after me very well. I'm quite happy to consult with him on what I'm doing. I think three, four, I don't know maybe even five occasions [in the last 6 years] I've had urinary infection [smell and lesser feeling of well-being], he gives me antibiotics straight away and they disappear in no time at all. (Participant 10, male)

Another emphasised the importance of seeing the same GP who '*understands what I've been going through*' and '*knows my history'* :

…he understands, he knows what I've been through, when you have to explain it to somebody else, it's all there in the notes, whether they look back in anything, but you have to keep explaining things, whereas Dr [name] understands what I've been going through. (Participant 23, female)

3 UTI prevention and understanding the causes

3.1 Clean/correct technique

Many attributed UTI to faulty IC technique - for example, accidently touching the catheter or not washing hands well enough, but were not sure what they could do differently to prevent a UTI:

I've got [infections no more than twice a year], obviously didn't do it well enough. It's not good enough really, I mean soap is good and is good, perhaps I should leave it longer, perhaps the [name of disinfectant] should have longer to work, I don't know, I really don't know, I don't know what the time is for [name of disinfectant]. (Participant 28, male)

Public toilets were seen as a potential cause, not only because such environments are perceived as a source of infection, but also because it was more difficult to carry out IC correctly. For one man, his decision to avoid public toilets limited his outings:

For peer review only - http://bmjopen.bmj.com/site/about/guidelines.xhtml

The problem was when I first started catheterising and going out, I was still getting infections from using public toilets things like that, so I decided I'd change the way I did things and I only go out for half a day now. So I come back midday. So I try not to catheterise when I'm out. (Participant 40, male)

3.2 Emptying bladder completely

Maintaining a regular schedule of bladder emptying was considered an important preventative strategy. One participant noted:

In the early stages [I had infections] every couple of weeks and it was a bit worrying at first. […] Sometimes I wouldn't [catheterise] twice a day, I'd only do it once. I realised that I've got to do it twice a day. […] I think making sure I do it twice a day and making sure everything is sterile. (Participant 1, male)

3.3 Good diet and lifestyle

Good diet and lifestyle was another prevention strategy - no alcohol, caffeine or sugar plus walking were seen as healthy habits to help prevent UTI:

To try and prevent infection, I don't drink alcohol. Not for 3 years. I don't have caffeine. I was concerned that I was taking chocolate as an energy boost but I am really limiting anything with it; I don't have sugar in anything, I've not had sugar in anything for about 35 years. But I was having the odd biscuit and chocolate and the intake was increasing and I was thinking 'hold on, this has got to stop' so I try and do things that will prevent infection anyway. I drink quite a lot of water. […]I have a walk about 20 minutes each day. (Participant 41, male)

For peer review only - http://bmjopen.bmj.com/site/about/guidelines.xhtml

DISCUSSION

The study shows that there is uncertainty among IC users about signs and symptoms of UTI and when to seek help; furthermore, individual descriptions of UTI often fit only generally within the NIDRR descriptors and may require more user friendly language. The non-specific nature of many UTI signs and symptoms presents a challenge for the GP, who relies on the IC user's description, together with urine culture results, to determine the most appropriate course of action.[2] Cloudy or smelly urine was the most frequently cited indicator and whilst some acted on this alone and visited the GP, others described how they would first attempt to self-manage and monitor for further symptoms, such as localised pain, discomfort or generalised illness. Some with co-morbidities had difficulty separating possible UTI from other more general signs and symptoms. Of note is that although 50% of participants described some change in bladder function (e.g. frequency), this was not included in the NIDRR symptom list. This is most likely because the NIDRR descriptors were focused on individuals with neurogenic impairment as a result of SCI. Long-term IC users vary widely and many have urethral sensation so that frequency and urgency could be expected as symptoms. The language used to describe the signs and symptoms may differ from typical clinically accepted terms. Comprehension of terminology surrounding IC could be a focus for further research and help equip new IC users with the necessary confidence to advocate for themselves or to manage symptoms. The decision to seek medical assistance was influenced by (1) an individual's perception of symptom severity, (2) the level of confidence in the effectiveness of self-help strategies, and (3) the previous experience of UTI and its management by the GP.

The key role of the GP in helping the individual manage, whether with symptom-based treatment or having a repeat prescription, was seen as invaluable. Although some of our participants were uncertain about the symptoms of UTI, in general people using IC on a long

For peer review only - http://bmjopen.bmj.com/site/about/guidelines.xhtml

term basis become very familiar with the warning signs of health issues and want to be proactive in their care. In this study, IC users indicated management was facilitated by the positive relationship and continuity with their GP who was aware of the individual's health status and approached care on an individual basis. This 'interdependence' of the individual and the physician is crucial and the reliance alone on laboratory testing should not define treatment in this population.[3] That self-medication with antibiotics might help IC users feel more confident and secure about managing symptoms needs further exploration.

Finally, health care professionals need to be attuned to individuals who self blame ('not hygienic enough') or lack an understanding of the aetiology of UTI. Such feelings were highlighted in at least one other study involving women with non-neurogenic dysfunction where women attributed UTI to 'poor hygiene', 'negligence', 'not drinking enough' or a 'penalty of growing old'.[13] In our study, some participants unnecessarily restricted their lives by following self-imposed rigid and complex procedures (i.e., not going out all day so they could catheterise only at home; restricting their diets; never using public toilets). When an IC user approaches the GP for assistance, exploring the story and probing for the effect of IC on daily life aids in making a collaborative plan for evidence based self-care. This requires the IC users to understand signs and symptoms which are relevant for them and to have the language to articulate their concerns to the GP. Many of the individuals in our study had used IC for several years and might have benefited from an updated educational session on current best practice with IC, causes of UTI, and use of antibiotics.

Strengths and limitations

This is the first qualitative study to explore views and experiences of UTI from a focussed IC user perspective. A strength is the inclusion of IC users with a range of reasons for use, including both neurological or non-neurological conditions. In common with other qualitative

For peer review only - http://bmjopen.bmj.com/site/about/guidelines.xhtml

research, collecting data through interviews relies on recall of experiences, and our sample does not represent all IC users. All descriptions of UTI discussed were subjective and self-reported. Diagnoses were not confirmed so that described signs and symptoms may not have been caused by UTI. Participants talked generally about their antibiotic use for UTI treatment; however, the interview schedule included no questions specifically related to attitudes to or understanding of antibiotics or the evidence related to UTI and intermittent catheterisation. Advice such as use of urine dipsticks was not confirmed and may have been a misrepresentation of what the individual was actually taught.

Implications for future research or clinical practice

To address the most appropriate course of action regarding UTI in IC users, there is a need to develop a more patient-based UTI symptom list as well as an evidence-based algorithm for self-care and help seeking. Further exploration of the subjective descriptions of UTI signs and symptoms and matching these with laboratory confirmation may aid both the IC user and the GP in providing best care. A simple, evidence-based list of questions in lay terms (Table 2) could be used by IC users or health care professionals as a prompt to collect signs and symptoms and may assist in self-management and appropriate antibiotic use in this popoulation.

Table 2: Proposed set of questions to collect subjective descriptions of UTI symptoms from IC users

Symptoms	Prompting questions	If yes,
Changes to urine	Has your urine changed in colour?	How has it changed?
	Has your urine changed in smell?	How has it changed?
	Have you seen any specs in your urine?	
Changes in bladder emptying	Have you had any changes in bladder emptying?	How has it changed?

For peer review only - http://bmjopen.bmj.com/site/about/guidelines.xhtml

	Are you leaking urine more than usual?	
General symptoms	Do you feel unwell?<br>Do you feel more tired than usual?	How do you feel?
	Are you feeling hot or feverish?	
Pain/discomfort	Do you have any pain or discomfort ?	Where do you have the pain/discomfort?<br>(your back, tummy, bladder or to passing urine)<br>How do you feel?

CONCLUSION

The study has provided insight into how participants construct their experience of UTI and identified some opportunities for further study particularly related to on-going IC user education and follow up, antibiotic use, and self-care practices. Current guidelines[14] state that cloudy or malodorous urine in the catheterised adult (including IC users) should not be used alone to differentiate asymptomatic bacteriuria from infection or as an indication for urine culture or antimicrobial therapy, yet in the current study these were the most common changes reported by IC users and the impetus for help-seeking.

Focussed follow up according to evidence-based guidelines may assist in self-management and appropriate antibiotic use. Examining GP's perspectives would provide a balanced approach to improve the diagnosis and management of UTI amongst IC users. This study provides the basis for developing a self-help tool which may aid identification of UTI and communication with health care professionals.

Acknowledgements

For peer review only - http://bmjopen.bmj.com/site/about/guidelines.xhtml

We would like to thank all the interviewees who agreed to take part and share their experiences.

We thank also Samantha Sartain, PhD and Bridget Clancy, RGN, BSc for conducting part of the data collection, and Margaret Macaulay, RGN MSc for assistance with the manuscript.

Contributors

MF was the principal investigator and was primarily responsible for the original grant application. MF, JP, and MA were involved in designing the study and developing the methods. JP led the overall study team. SS collected the majority of the data. BC and MA also contributed to the data collection. IO led the analysis and interpretation of the qualitative data, and wrote drafts of the manuscripts. IO, JP, MA, KM and MF wrote drafts and critically revised the manuscripts.

Funding: This paper refers to independent research funded by the National Institute for Health Research (NIHR) under its Programme Grants for Applied Research (PGfAR) (Grant Reference Number RP-PG-0610-10078).

Disclaimer: The views expressed are those of the authors and not necessarily those of the NHS, the NIHR or the Department of Health.

Competing interests: None declared.

Ethical approval: Ethical permission was granted from a NHS Research Ethics Committee (NRES committee London Hampstead; REC reference 13/LO/1511; approval letter dated 2 October 2013).

Provenance and peer review: Not commissioned; externally peer reviewed.

Data sharing statement: No additional data are available.

For peer review only

References

1. Wyndaele JJ. Complications of intermittent catheterization: Their prevention and treatment. *Spinal Cord* 2002;40(10):536-41.
2. Wyndaele JJ, Brauner A, Geerlings SE, et al. Clean intermittent catheterization and urinary tract infection: Review and guide for future research. *BJU Int* 2012;110(11 Pt C):E910-7.
3. The prevention and management of urinary tract infections among people with spinal cord injuries. National institute on disability and rehabilitation research consensus statement. January 27-29, 1992. *J Am Paraplegia Soc* 1992;15(3):194-204.
4. García Leoni ME, Esclarín De Ruz A. Management of urinary tract infection in patients with spinal cord injuries. *Clin Microbiol Infect* 2003;9(8):780-85.
5. Massa LM, Hoffman JM, Cardenas DD. Validity, accuracy, and predictive value of urinary tract infection signs and symptoms in individuals with spinal cord injury on intermittent catheterization. *J Spinal Cord Med* 2009;32(5):568-73.
6. Cobussen-Boekhorst H, Hermeling E, Heesakkers J, et al. Patients' experience with intermittent catheterisation in everyday life. *J Clin Nurs* 2016;25(9-10):1253-61.
7. Kelly L, Spencer S, Barrett G. Using intermittent self-catheters: Experiences of people with neurological damage to their spinal cord. *Disabil Rehabil* 2014;36(3):220-26.
8. Shaw C, Logan K. Psychological coping with intermittent self-catheterisation (isc) in people with spinal injury: A qualitative study. *Int J Nurs Stud*;50(10):1341-50.
9. Wilde MH, Brasch J, Zhang Y. A qualitative descriptive study of self-management issues in people with long-term intermittent urinary catheters. *J Adv Nurs* 2011;67(6):1254-63.
10. Logan K, Shaw C. Intermittent self-catheterization service provision: Perspectives of people with spinal cord injury. *International Journal of Urological Nursing* 2011;5(2):73-82.
11. Jaquet A, Eiskjaer J, Steffensen K, et al. Coping with clean intermittent catherization--experiences from a patient perspective. *Scand J Caring Sci* 2009;23(4):660-6.
12. Logan K, Shaw C, Webber I, et al. Patients' experiences of learning clean intermittent self-catheterization: A qualitative study. *J Adv Nurs* 2008;62(1):32-40.
13. Leydon GM, Turner S, Smith H, et al. Women's views about management and cause of urinary tract infection: Qualitative interview study. *BMJ* 2010;340:c279.
14. Hooton TM, Bradley SF, Cardenas DD, et al. Diagnosis, prevention, and treatment of catheter-associated urinary tract infection in adults: 2009 international clinical practice guidelines from the infectious diseases society of america. *Clin Infect Dis* 2010;50(5):625-63.

For peer review only

Table 1: Signs and symptoms of UTI described and mapped to NIDRR consensus statement

	**Perceived signs and symptoms of UTI described by IC users**	**Mapping to the NIDRR consensus statement**	**N (%) Total=30**
Changes to urine	• Different colour<br>• Cloudiness<br>• Darkness in the urine<br>• A bit like rather thick soup	Cloudy urine	24 (80%)
	• The smell of urine not being right<br>• A really strong smell<br>• Smell<br>• Horrible smell<br>• Change in the odour of the urine	Urine with increased odour	22(73%)
	• Looked like there was a little bit of blood present<br>• Specks of blood		4 (13%)
	• There was protein present (found at GP)	Leukocytes in the urine generated by the mucosal lining	2 (7%)
Changes in bladder emptying	• Feel like you need to go more often but not passing enough<br>• I was going to the toilet, and then not feeling as I'd actually been; there was still a pressure there of wanting to go<br>• You feel you want to keep having a pee constantly<br>• Having to go more frequently<br>• Can't pass water properly<br>• Be able to have a normal pee without having to use a catheter<br>• I feel as though I empty my bladder continually<br>• Going to toilet less		15 (50%)
	• Leak a bit between catheterisation<br>• Cannot hold my water; a couple of accidents during the day	Onset of urinary incontinence	4 (13%)
General symptoms	• Not feeling well<br>• I feel low, very rundown<br>• don't feel right<br>• lesser feeling of well-being; slight feeling of discomfort, not quite right<br>• don't feel comfortable	Malaise, lethargy	12 (40%)

For peer review only

For peer review only - http://bmjopen.bmj.com/site/about/guidelines.xhtml

	• Hot and sticky<br>• Bad fever, high temperature<br>• Very hot and shivery, not much of a temperature<br>• A fever symptom	fever	8 (27%)
	• I feel like I don't want to go anywhere and I feel tired<br>• In terms of worrying about needing to go to the loo a lot more<br>• I just worried about (UTI)<br>• Don't feel right in yourself	Sense of unease	4 (13%)
	• Vertigo<br>• Felt as if I was going to pass out<br>• Couldn't eat anything; constantly being sick; diarrhoea<br>• Devastating		4 (13%)
Pain/ discomfort	*Discomfort or pain over bladder*<br>• The bladder gets very irritated; the whole pelvis is quite irritated<br>• Burning bladder<br>• The pain usually starts in the urethra area<br>• Slight irritation (in my urethra and around that area)	Discomfort or pain over the kidney or bladder, or during urination	9 (30%)
	Discomfort or pain over kidney<br>• tremendous pain in my back, in my kidney area<br>• dull ache in my lower back and stinging		3 (10%)
	Pain during urination<br>• Get a pain when you are having a pee<br>• Burning when I pass urine<br>• Very difficult to pass water<br>• When I go to the loo, it slightly burns		5 (17%)
	Discomfort or pain in penis/groin area<br>• Stinging feeling in your penis itself		6(20%)
	Non-localised discomfort/pain<br>• Feel uncomfortable down below<br>• Get uncomfortable; when I sit down, feels like there is something there; uncomfortable to walk<br>• It's really really painful<br>• I wouldn't say painful, but it's		3 (10%)

For peer review only

	discomfort<br>• Pain, stinging sometimes<br>• Feeling uncomfortable; even if I've used a catheter, still feels uncomfortable<br>• It burns like mad. There's no part that doesn't burn<br>• a tenderness all around the area<br>• everything down there feels very irritable; feel very uncomfortable<br>• it gets extremely painful		
	Not reported	Increased spasticity	
	Not reported	Autonomic dysreflexia	

For peer review only

MULTICATH MODULE 2 SCHEDULE FOR ITERATION 3

Background questions

1. Experience of intermittent catheterisation:

    How long have you been using intermittent catheterisation?

    Do you self-catheterise? Or does somebody help you?

    How many times per day do you catheterise?

    What catheter do you use?

    Do you catheterise:

        At home only?

        When you are out?

        If yes, when you are out what kind of catheter do you use, is it the same one as for home use?

Beliefs and practices re IC routine

2. Beliefs and concerns about IC rituals, catheters, infection and sterility

    What steps do you take before you do IC?

    What is the reason you take these steps? (Probe without leading for whether or not they value sterility)

    Is your IC routine clean or sterile?

    What is sterile to you? (prompt – how is it different to clean?) What do you mean by clean?

Experience of urine infection

3. During your time using intermittent catheterisation have you ever experienced a urine infection?

    If yes, how often would you say you have a urine infection?

    Can you recall the last time you had a UTI? What happened?

4. What it feels like to have a urine infection:

    Can you describe how you feel when you think you may be developing a urine infection?

    Does anything happen beforehand when you think one is coming on?

For peer review only

For peer review only - http://bmjopen.bmj.com/site/about/guidelines.xhtml

How do you think your infection came about? Prompt (Where do you think the germs come from when you get a UTI?)

What do you do to prevent an infection?

What advice do you get/have you had from your health care professional to prevent infections?

Complete the table – UTI signs and symptoms: Where patients name a symptom or sign check with them the terminology and that the patient is happy that it sits within a category on the table. We want to understand the terminology that patients use to describe UTI symptoms.

Looking at the symptoms listed here, are these the right things that describe a urine infection from your experience? Is anything missing?

5. Seeking help:

When you feel you may have a urine infection, what do you do about it?

Prompt: antibiotics

At what point do you seek help from others?

Who do you seek help from? And what do you do?

Cleaning questions

6. Methods of cleaning:

Have you ever re-used your catheter?

If Yes, Under what circumstances? Did you clean it after its first use? How did you clean it? How did you store it between uses?

What is/would be acceptable to you?

If No, Have you ever thought about re-using your catheters?

If you were offered a method of cleaning and re-using your catheters instead of using a new one each time, what kinds of things would be important to you? [Prompts: How to clean; Where to clean; Where to store between uses]

What kind of cleaning regime is acceptable to you? [Prompts: what cleaning methods and products, what about storage storage/travelling, and lubrication?]

What would worry you about re-using your catheter? [Prompts: cleaning in a public loo, being at work, storing and transporting]

What would be good about re-using?

For peer review only

Discrete choice experiment questions

[I would now like to discuss with you the benefits and drawbacks of using disposable and reusable

Catheters]

7. What is good about your current catheter?

What do you think about single-use catheters?

What are the benefits of using a disposable or single-use catheter?

What are the drawbacks?

[in the following situations]

- At home?
- At work?
- On holiday?
- In a public place?
- Going out?
- Other?

What do you think about the idea of a re-usable catheter?

- What do you see as a benefit of re-using your catheter?
- What do you see as a drawback in re-using your catheter?

[in the following situations]

- At home?
- At work?
- On holiday?
- In a public place?
- Going out?
- Other?

- What would encourage you to use it?
- What would discourage you from using it?

Preamble: We know from previous interviews/speaking to other patients, that avoidance of infection is what matters most to everyone. We also know that other things are highly important such as:

- a comfortable insertion,
- having a catheter that is easy to use and handle,
- that comes in packaging which is easy to open,
- that your catheter is discrete,
- that you can trust your catheter/ trust in the product

As we already know all of this, we now want to explore other things around single-use catheters and re-usable catheters.

For peer review only - http://bmjopen.bmj.com/site/about/guidelines.xhtml

Together let's go through each item in this list and tell me what you think about each one? Your comments are very valuable to us.

Those relating to disposable catheters:

1. That I don't have the bother of cleaning the catheter
2. That I don't need to have special equipment for cleaning the catheter
3. That I don't have to carry used/soiled catheters around with me in my bag

Those relating to re-using catheters:

4. That I have less catheters to carry with me on holiday
5. That my catheters don't cost the NHS more than they need to/too much
6. That I don't have to store lots of catheters at home
7. That I don't have to worry that I will run out of catheters
8. That I don't have to get rid of lots of rubbish when I am out
9. That my catheters are not bad for the environment.

For peer review only

For peer review only - http://bmjopen.bmj.com/site/about/guidelines.xhtml

The MultICath Study (LREC: 13/LO/1511) - Urinary Tract Infection (UTI) signs and symptoms

Participant study ID: ______________ **Date of completion:** _______________ Completed by (interviewer name): ___________________

Tick all the signs and symptoms that refer to your experience of UTI:

**Signs and symptoms**													
	**Autonomic dysreflexia**	**Fever**	**Kidney /Bladder discomfort**	**Increased Bladder activity (spasticity)**	**Feeling sick**	**Sense of unease**	**Increased catheterising**	**Feeling tired**	**Incontinence**	**Foul smelling urine**	**Cloudy urine**	**White blood cells in urine**	**Haematuria**
Tick if yes													
**Circle how often you experience each symptom when you get a UTI**													
Always													
Sometimes													
Rarely													

Which of the above symptoms they mentioned would cause them to seek help from GP? _________________________

Which of the above symptoms they mentioned would cause them to take antibiotics (patients with own supply)? __________________________

How often does the patient have a UTI? (tick which applies)

**Once a week**	**Once a month**	**Every other month**	**Twice a year**	**Once a year**	**Less than once a year**

How many times has the patient been to their GP in the last year for a UTI? ______________________________

How many times has the patient taken antibiotics in the last year for a UTI?______________________________

Have you been referred to a specialist in the last year for UTI symptoms? (eg. Urologist) ____________________

For peer review only

COREQ (COnsolidated criteria for REporting Qualitative research) Checklist

A checklist of items that should be included in reports of qualitative research. You must report the page number in your manuscript where you consider each of the items listed in this checklist. If you have not included this information, either revise your manuscript accordingly before submitting or note N/A.

Topic	Item No.	Guide Questions/Description	Reported on Page No.
**Domain 1: Research team and reflexivity**			
Personal characteristics			
Interviewer/facilitator	1	Which author/s conducted the interview or focus group?	
Credentials	2	What were the researcher's credentials? E.g. PhD, MD	
Occupation	3	What was their occupation at the time of the study?	
Gender	4	Was the researcher male or female?	
Experience and training	5	What experience or training did the researcher have?	
Relationship with participants			
Relationship established	6	Was a relationship established prior to study commencement?	
Participant knowledge of the interviewer	7	What did the participants know about the researcher? e.g. personal goals, reasons for doing the research	
Interviewer characteristics	8	What characteristics were reported about the inter viewer/facilitator? e.g. Bias, assumptions, reasons and interests in the research topic	
**Domain 2: Study design**			
Theoretical framework			
Methodological orientation and Theory	9	What methodological orientation was stated to underpin the study? e.g. grounded theory, discourse analysis, ethnography, phenomenology, content analysis	
Participant selection			
Sampling	10	How were participants selected? e.g. purposive, convenience, consecutive, snowball	
Method of approach	11	How were participants approached? e.g. face-to-face, telephone, mail, email	
Sample size	12	How many participants were in the study?	
Non-participation	13	How many people refused to participate or dropped out? Reasons?	
Setting			
Setting of data collection	14	Where was the data collected? e.g. home, clinic, workplace	
Presence of non-participants	15	Was anyone else present besides the participants and researchers?	
Description of sample	16	What are the important characteristics of the sample? e.g. demographic data, date	
Data collection			
Interview guide	17	Were questions, prompts, guides provided by the authors? Was it pilot tested?	
Repeat interviews	18	Were repeat inter views carried out? If yes, how many?	
Audio/visual recording	19	Did the research use audio or visual recording to collect the data?	
Field notes	20	Were field notes made during and/or after the inter view or focus group?	
Duration	21	What was the duration of the inter views or focus group?	
Data saturation	22	Was data saturation discussed?	
Transcripts returned	23	Were transcripts returned to participants for comment and/or	

For peer review only

Topic	Item No.	Guide Questions/Description	Reported on Page No.
		correction?	
**Domain 3: analysis and findings**			
Data analysis			
Number of data coders	24	How many data coders coded the data?	
Description of the coding tree	25	Did authors provide a description of the coding tree?	
Derivation of themes	26	Were themes identified in advance or derived from the data?	
Software	27	What software, if applicable, was used to manage the data?	
Participant checking	28	Did participants provide feedback on the findings?	
Reporting			
Quotations presented	29	Were participant quotations presented to illustrate the themes/findings? Was each quotation identified? e.g. participant number	
Data and findings consistent	30	Was there consistency between the data presented and the findings?	
Clarity of major themes	31	Were major themes clearly presented in the findings?	
Clarity of minor themes	32	Is there a description of diverse cases or discussion of minor themes?	

Developed from: Tong A, Sainsbury P, Craig J. Consolidated criteria for reporting qualitative research (COREQ): a 32-item checklist for interviews and focus groups. *International Journal for Quality in Health Care*. 2007. Volume 19, Number 6: pp. 349 – 357

Once you have completed this checklist, please save a copy and upload it as part of your submission. DO NOT include this checklist as part of the main manuscript document. It must be uploaded as a separate file.

For peer review only

BMJ Open

Intermittent catheter users' symptom identification, description, and management of urinary tract infection: a qualitative study

Journal:	*BMJ Open*
Manuscript ID	bmjopen-2017-016453.R1
Article Type:	Research
Date Submitted by the Author:	09-Jun-2017
Complete List of Authors:	Okamoto, Ikumi; University of Southampton, Faculty of Health Sciences<br>Prieto, Jacqui; University of Southampton, Faculty of Health Sciences<br>Avery, Miriam; University of Southampton, Faculty of Health Sciences<br>Moore, Katherine ; University of Alberta<br>Fader, Mandy; University of Southampton, Faculty of Health Sciences
**Primary Subject Heading**:	Urology
Secondary Subject Heading:	Urology, Qualitative research
Keywords:	Urinary tract infections < UROLOGY, QUALITATIVE RESEARCH, PRIMARY CARE

SCHOLARONE™ Manuscripts

For peer review only

For peer review only - http://bmjopen.bmj.com/site/about/guidelines.xhtml

Intermittent catheter users' symptom identification, description, and management of urinary tract infection: a qualitative study

Corresponding author
Mandy Fader
Faculty of Health Sciences, University of Southampton, South Academic Block (MP11), Southampton General Hospital, Tremona Road, Southampton SO16 6YD
m.fader@soton.ac.uk
(023) 8059 6677

Authors
Ikumi Okamoto[1]
Jacqui Prieto[1]
Miriam R. Avery[1]
Katherine N. Moore[2]
Mandy Fader[1]

[1]Faculty of Health Sciences, University of Southampton, South Academic Block (MP11), Southampton General Hospital, Tremona Road, Southampton SO16 6YD
[2] Faculty of Nursing, University of Alberta, Edmonton, Canada

Word count

3834 words at the moment (from Intro to Conclusion excluding tables)

For peer review only

For peer review only - http://bmjopen.bmj.com/site/about/guidelines.xhtml

ABSTRACT

Objectives
To elucidate the views of intermittent catheter (IC) users regarding urinary tract infection (UTI) symptom presentation, terminology for describing signs and symptoms, the cause of UTI and management strategies.

Design
Qualitative study with semi-structured interviews. The transcribed text was analysed thematically.

Setting
12 GP surgeries in Hampshire and Dorset, UK.

Participants
A convenience sample of 30 IC users; aged over 18, using IC for at least 3 months who had at least one self-reported UTI since starting IC.

Results
Participants reported a variety of signs and symptoms, such as urine cloudiness and smell, as indicators of UTI. The terms used often differed from those in the modified National Institute on Disability and Rehabilitation Research (NIDRR) symptom set. IC users had difficulty distinguishing possible UTI symptoms from those of their co-morbidities. They expressed uncertainty about the cause of UTI, often attributing it to poor hygiene and lifestyle behaviours. Whereas some viewed UTI as an expected consequence of IC use that could be self-managed, others felt more concerned and were more reliant on their General Practitioner (GP) for support. A range of management strategies were described, including drinking more fluids, increased attention to personal hygiene and self-medicating with antibiotics.

Conclusions
There is uncertainty among IC users about UTI signs and symptoms and when to seek help. Individual accounts of UTI fitted generally within the modified NIDRR descriptors but adopted less technical and more ‘lay’ language. IC users’ descriptions of UTI signs and symptoms can lack precision, owing partly to the presence of underlying health conditions. This, together with differing levels of concern about the need to seek help and self-medication with antibiotics, presents challenges for the GP. This study provides the basis for developing a self-help tool which may aid identification of UTI and enhance communication with health care professionals.

Strengths and limitations of this study

- This is the first qualitative study to explore IC users’ views, experiences, and descriptions of UTI signs and symptoms.
- The study includes IC users who had a wide range of reasons for using IC, including both neurological or non-neurological conditions.
- All UTI signs and symptoms reported in this paper were subjective, retrospective, and self-reported, and were not confirmed by laboratory analysis.
- Advice from heatlh care professionals that IC users discussed was not confirmed.

For peer review only

INTRODUCTION

Intermittent catheterisation (IC) is widely used for the urological management of people with incomplete bladder emptying.[1 2] Urinary tract infection (UTI) is the most frequently reported and challenging complication.[2] In 1992 medical experts developed a key document on diagnosis and management of UTI in spinal cord injured individuals (SCI) for the National Institute on Disability and Rehabilitation Research (NIDRR).[3] The signs and symptoms listed in 1992 remain the current clinical standard to guide practice for the neurogenic patient: positive urine culture plus one or more signs or symptoms (leukocytes in the urine, discomfort or pain over the kidney or bladder or during voiding, onset of urinary incontinence, fever, increased spasticity, autonomic dysreflexia, cloudy malodorous urine, or malaise, lethargy, sense of unease). However, the document did not include patient contributions from non-SCI IC users to the terminology nor to the presenting symptoms and therefore cannot be considered to fully reflect the experience of the wider IC population. As UTI signs and symptoms can be difficult for some IC users to interpret, the NIDRR descriptors may be perceived differently or may not directly apply [2 4] and thus can be difficult for an individual to describe to their general practitioner (GP). Massa et al [5] used a UTI signs and symptoms list modified from the NIDRR criteria [3] with SCI individuals using IC, and found that only 66% could self-predict their own UTI. Research exploring how IC users themselves identify and describe the signs and symptoms of UTI, as compared to the existing criteria, may aid in understanding how these criteria are interpreted and when assistance is sought. Patient-oriented language may also assist in mutual understanding of the presence of a UTI.

Although there are studies exploring experiences and issues related to IC,[6-12] UTI has not been a focus. We found no qualitative research specifically exploring IC users' descriptions of UTI signs and symptoms nor how these descriptions compare to the existing symptom list. Thus in

For peer review only - http://bmjopen.bmj.com/site/about/guidelines.xhtml

this study, we conducted qualitative interviews with IC users with the aim of investigating their descriptions of UTI signs and symptoms, including their strategies for prevention, identification, and management, with the goal of informing further research on a symptom-specific tool for IC users.

METHOD

The study used a qualitative method. Convenience sampling was used to recruit participants using IC on a regular basis from 12 GP practices in Hampshire and Dorset, UK to take part in interviews regarding their experiences of IC as part of a larger study (the MultiCath programme[13]). Participants were over 18 years of age, had independently used IC for at least 3 months, and had no reported urethral deformities or immune deficiency disease. Ethical approval was obtained from the appropriate local research ethics committees. Written consent was obtained from all participants.

Data collection

One face to face interview lasting 45-60 minutes was conducted in each participant's home by one of three experienced female interviewers including a qualitative researcher (SS) and research nurses (BC and MA). None of the interviewers had had previous contact with any of the participants, and they attended as a non-clinical and non-judgemental listener. If desired, a relative or carer of the participant was present. A semi-structured interview schedule (Appendix 1) was used to explore experiences, values, beliefs and themes around the use of catheters generally. Based on UTI related symptoms identified from the first 6 interviews, we then added haematuria to the modified NIDRR symptom list [5] (Appendix 2) . The list was then used in subsequent interviews. Participants were prompted to describe their experience of UTI signs and symptoms in their own words. They were also shown the symptom list to go through with the interviewer, which helped participants recall further signs and symptoms they had

For peer review only - http://bmjopen.bmj.com/site/about/guidelines.xhtml

experienced. Recruitment continued until data saturation was reached. Interviews were recorded and then transcribed verbatim. All transcripts were anonymised with an assigned study number. The transcripts were read only by the researchers and not by participants. Field notes were made during the interviews, which were used in the analysis.

Data analysis

A qualitative researcher (IO), who had not conducted the interviews, analysed the data thematically and coded it using NVivo 10 (QSR International). The first 20 transcripts formed an initial coding framework. Coding was further refined through discussion with the wider research team and ongoing analysis of the remaining transcripts. Newly emerged themes that did not fit into the initial themes were discussed as a team. No participants were involved in the analysis of data.

RESULTS

Participants

One hundred and thirty-nine IC users were invited to take part and 74 (53%) responded, of whom 42 were willing to participate. Three were ineligible. Nine participants had never experienced a UTI since starting IC, and were not asked any further questions regarding UTI experiences during the interview. Therefore, the findings presented here are based on a sub-set of our sample. The total number of participants was 30, including 19 men and 11 women, mean age 66 years (range 23-86), using IC for approximately 10 years (range 9 months – 31 years). The frequency of IC ranged from 1-10 catheterisations a day (mean 4/day). Reasons for IC included chronic urinary retention (N=15), neurological impairment (N=10), and other factors such as post surgery or post chemotherapy (N=5). Three were currently re-using their catheters but as they were few in number, no distinction was made between re-users and single-users

For peer review only - http://bmjopen.bmj.com/site/about/guidelines.xhtml

during data analysis. All participants were English speakers. No race/ethnicity data were collected.

Findings

Three themes were identified regarding experiences of UTI: 1) IC users' description of their signs and symptoms and degree of certainty; 2) help-seeking actions when UTI is suspected; and 3) understanding of causation and prevention strategies used.

1 IC users' description of their signs and symptoms and degree of certainty

1.1 Terms used to describe signs and symptoms

The participants reported a variety of signs and symptoms that they thought were caused by UTI. Table 1 provides examples of participants' descriptions of the UTI signs and symptoms collected during the interviews with a corresponding comparison to the modified NIDRR criteria[5]. Participants were likely to use informal terms to describe their signs and symptoms such as 'cannot hold my water' or 'having to go more frequently' rather than 'incontinence' and 'increased catheterising' respectively (for more examples see Table 1). If they described pain it was often generalised and not localised until they were prompted. Terms such as 'discomfort' and 'uncomfortable' were used to describe both general physical symptoms and pain or even increased urinary frequency – '*everything feels very irritable … in the area of the penis or the bladder. You just feel very uncomfortable …as if you want to go all the time' (Particpant 41, male)*. Corresponding terms in the modified NIDRR set such as 'discomfort or pain over the kidney or bladder' did not cover the breadth of symptoms experienced. The language used to describe the signs and symptoms often differed from typical clinically accepted terms; however, none of the participants voiced concern about being unable to communicate effectively with GPs.

For peer review only - http://bmjopen.bmj.com/site/about/guidelines.xhtml

Table 1: Participants' descriptions of UTI signs and symptoms and mapped to modified NIDRR symptom list

Symptom categories	Examples of participants' descriptions of UTI signs and symptoms collected during the interviews	Mapping to the modified NIDRR symptom list [5]
Changes to urine	• Different colour<br>• Cloudiness<br>• Darkness in the urine<br>• A bit like rather thick soup	Cloudy urine
	• The smell of urine not being right<br>• A really strong smell<br>• Smell<br>• Horrible smell<br>• Change in the odour of the urine	Foul smelling urine
	• Looked like there was a little bit of blood present<br>• Specks of blood	Haematuria [*]
	• There was protein present (found at GP)	Leukocytes in the urine
Changes in bladder emptying	• Feel like you need to go more often but not passing enough<br>• I was going to the toilet, and then not feeling as I'd actually been; there was still a pressure there of wanting to go<br>• You feel you want to keep having a pee constantly<br>• Having to go more frequently<br>• Can't pass water properly<br>• Be able to have a normal pee without having to use a catheter<br>• I feel as though I empty my bladder continually<br>• I've got to do [IC] twice as much<br>• Going to toilet less	Increased frequency of catheterisation
	• Leak a bit between catheterisation<br>• Cannot hold my water; a couple of accidents during the day	Incontinence
Fever	• Hot and sticky<br>• Bad fever, high temperature<br>• Very hot and shivery, not much of a temperature	Fever

For peer review only - http://bmjopen.bmj.com/site/about/guidelines.xhtml

	• A fever symptom	
Generalised/ Systemic Symptoms	• Not feeling well<br>• I feel low, very rundown<br>• Don't feel right<br>• Lesser feeling of well-being; slight feeling of discomfort, not quite right<br>• Don't feel comfortable	Feeling tired
	• I feel like I don't want to go anywhere and I feel tired<br>• Don't feel right in yourself	Sense of unease
	• Vertigo<br>• Felt as if I was going to pass out<br>• Couldn't eat anything; constantly being sick; diarrhoea	Feeling sick
Pain/ discomfort	*Discomfort or pain over bladder*<br>• The bladder gets very irritated; the whole pelvis is quite irritated<br>• Burning bladder<br>• The pain usually starts in the urethra area<br>• Slight irritation (in my urethra and around that area)	Discomfort or pain over the kidney or bladder
	Discomfort or pain over kidney<br>• Tremendous pain in my back, in my kidney area<br>• Dull ache in my lower back and stinging	
	Pain during urination<br>• Get a pain when you are having a pee<br>• Burning when I pass urine<br>• Very difficult to pass water<br>• When I go to the loo, it slightly burns	
	Discomfort or pain in penis/groin area<br>• Stinging feeling in your penis itself	

For peer review only

For peer review only - http://bmjopen.bmj.com/site/about/guidelines.xhtml

	Non-localised discomfort/pain<br>• Feel uncomfortable down below<br>• Get uncomfortable; when I sit down, feels like there is something there; uncomfortable to walk<br>• It's really really painful<br>• I wouldn't say painful, but it's discomfort<br>• Pain, stinging sometimes<br>• Feeling uncomfortable; even if I've used a catheter, still feels uncomfortable<br>• It burns like mad. There's no part that doesn't burn<br>• A tenderness all around the area<br>• Everything down there feels very irritable; feel very uncomfortable<br>• It gets extremely painful	
	Not reported	Autonomic dysreflexia
	Not reported	Increased spasticity

*An item added to the modified NIDRR list for our symptom list used during the interviews.

For peer review only

For peer review only - http://bmjopen.bmj.com/site/about/guidelines.xhtml

1.2 Uncertainty about whether symptoms were caused by UTI

Deciding if they had a UTI was difficult for some participants because of co-morbid conditions. For example, one with bladder cancer and "prostate problems" sometimes had a feeling of irritation but was unsure whether symptoms were caused by UTI or by his other health problems:

> *[Talking about UTI is] difficult because I've always had problems down below. I mean I [have] prostate problems, bladder problems, bladder cancer, a lot. So sometimes, I get this irritation feeling but [it] never bothers me. (Participant 12, male)*

Others also mentioned the difficulty of distinguishing UTI symptoms from their age-related problems.

> *I'm eighty-four and it's one of those things, you expect things to happen as you're getting old. Aches and pains, and stuff like that, it could be to do with that, I don't know, but you get so many bits and pieces. (Participant 4, male)*

2 Help-seeking actions when UTI is suspected

2.1 Decision process whether to seek help or not

Many participants reported that cloudy or smelly urine were the first indicators; however, how they managed such signs varied considerably. Most usually waited a few days to be convinced that their symptoms were caused by UTI before contacting their GP and used 'self-help' strategies such as increasing fluids:

For peer review only - http://bmjopen.bmj.com/site/about/guidelines.xhtml

[I] try to drink plenty and sort of flush it through and obviously if after a day or two I still felt bad or got worse I would have gone to the doctor's. (Participant 3, female)

Others waited until the onset of other symptoms, such as pain and discomfort, in addition to the first signs.

I think it's a little bit cloudy, but the next day it's perfectly clear. So I think one gets this in the way, sometimes, and if it is notably cloudy and I'm getting the pain [in the penis], it becomes pretty obvious that actually that's what I've got. (Participant 32, male)

Another stated:

Since [I started IC] I have had and may well even now have a urinary infection, but if I drink enough it just doesn't affect me. Would I want to be on antibiotics all the time? […] I think it is more important that [UTI] is not affecting your overall health. I probably have an infection at the moment but it doesn't affect me in any way, I don't have a temperature, I'm not ill as a result of it. I actually had it out with my doctor a bit because they did a test at one point and said that I had an infection and I must take antibiotics. I thought I can but I'll probably have another one in 3 or 6 months time. (Participant 37, male)

Conversely, others contacted the GP at the first sign, such as cloudy/smelly urine, as previous experience suggested that more severe symptoms would follow if left untreated:

I always know as soon as I've got [an infection] because as soon as it's there, the smell's there. And if I don't sort of act on it straight away … within a week

For peer review only - http://bmjopen.bmj.com/site/about/guidelines.xhtml

> *I'll be feeling really unwell. […] And if I leave it later, it takes a lot longer to get rid of it. (Participant 11, female)*

A participant's decision to consult their GP was also influenced by previous advice they had received about UTI. For example, a participant with cauda equina who '*lost most feeling below the waist*' said his GP had told him, *'You probably will know when you've got [UTI] if you get this sort of sickly-sweet smell.' (Pariticipant 10, male)*

2.2 Medication with antibiotics

If self-help strategies weren't effective, many contacted the GP for a prescription for antibiotics. There were three participants who self-medicated with antibiotics, which they kept at home. One felt that:

> *If you keep on having antibiotics that eventually it's not going to work. (Participant 13, male)*

For another, a supply of antibiotics was important as it reassured him that he had ready access to treatment:

> *It's funny, but if I had an infection, it's usually on a weekend, I can't catch anybody to get the antibiotics or whatever I need, so then I'd have to go to the hospital and all that palaver. So I just keep one in stock all the time. (Participant 19, male)*

> *[…] Sometimes [infection] will start by a burning in my willy [penis], and then it will back up further to the bladder, [but] I don't have that problems anymore, because I immediately put a pill into my mouth and off I go and three days later it's gone. (Participant 19, male)*

Seven took daily prophylactic antibiotics. One, using IC for 3 years, said:

For peer review only - http://bmjopen.bmj.com/site/about/guidelines.xhtml

For peer review only

I still get infections, off and on. You know when you get an infection, but I've taken tablets all the time for it. Every day, for infection. The doctor gave them to me, saying 'as soon as you're on a catheter, you've got to take these.' (Participant 4, male)

One participant mentioned that she used a urine dipstick test along with self administered antibiotics as suggested by the GP. She valued this approach and explained that it enabled her to act on a UTI promptly and avoid a trip to the GP surgery and risk seeing a physician who did not know her history:

You've got all the signs for a water infection [smell of urine, feeling hot and sticky, and leaking of urine], by the time you've got that appointment, you could have had that water infection for five days so you know what my old GP did? He sort of said you can go on the internet and you can buy the sticks to test your water yourself which we did, so I've got the sticks myself and my GP once a year tends to give me a prescription a repeat prescription for a couple of courses of antibiotics. (Participant 6, female)

2.3 Relationship with GPs

Having a good relationship with and easy access to the GP was greatly valued:

[My GP]'s obviously well aware of where I'm at and he looks after me very well. I'm quite happy to consult with him on what I'm doing. I think three, four, I don't know maybe even five occasions [in the last 6 years] I've had urinary infection [smell and lesser feeling of well-being], he gives me antibiotics straight away and they disappear in no time at all. (Participant 10, male)

For peer review only - http://bmjopen.bmj.com/site/about/guidelines.xhtml

For peer review only

Another emphasised the importance of seeing the same GP who '*understands what I've been going through*' and '*knows my history'* :

> *…he understands, he knows what I've been through, when you have to explain it to somebody else, it's all there in the notes, whether they look back in anything, but you have to keep explaining things, whereas Dr [name] understands what I've been going through. (Participant 23, female)*

3 Understanding of causation and prevention strategies used

3.1 Clean/correct technique

Many attributed UTI to faulty IC technique - for example, accidently touching the catheter or not washing hands well enough, but were not sure what they could do differently to prevent a UTI:

> *I've got [infections no more than twice a year], obviously didn't do it well enough. It's not good enough really, I mean soap is good and is good, perhaps I should leave it longer, perhaps the [name of disinfectant] should have longer to work, I don't know, I really don't know, I don't know what the time is for [name of disinfectant]. (Participant 28, male)*

Public toilets were seen as a potential cause, not only because such environments are perceived as a source of infection, but also because it was more difficult to carry out IC correctly. For one man, his decision to avoid public toilets limited his outings:

> *The problem was when I first started catheterising and going out, I was still getting infections from using public toilets things like that, so I decided I'd change the way I did things and I only go out for half a day now. So I come back midday. So I try not to catheterise when I'm out. (Participant 40, male)*

For peer review only - http://bmjopen.bmj.com/site/about/guidelines.xhtml

3.2 Emptying bladder completely

Maintaining a regular schedule of bladder emptying was considered an important preventative strategy. One participant noted:

> *In the early stages [I had infections] every couple of weeks and it was a bit worrying at first. […] Sometimes I wouldn't [catheterise] twice a day, I'd only do it once. I realised that I've got to do it twice a day. […] I think making sure I do it twice a day and making sure everything is sterile. (Participant 1, male)*

3.3 Good diet and lifestyle

Good diet and lifestyle was another prevention strategy - no alcohol, caffeine or sugar plus walking were seen as healthy habits to help prevent UTI:

> *To try and prevent infection, I don't drink alcohol. Not for 3 years. I don't have caffeine. I was concerned that I was taking chocolate as an energy boost but I am really limiting anything with it; I don't have sugar in anything, I've not had sugar in anything for about 35 years. But I was having the odd biscuit and chocolate and the intake was increasing and I was thinking 'hold on, this has got to stop' so I try and do things that will prevent infection anyway. I drink quite a lot of water. […] I have a walk about 20 minutes each day. (Participant 41, male)*

DISCUSSION

The study shows that there is uncertainty among IC users about signs and symptoms of UTI and when to seek help; furthermore, individual descriptions of UTI signs and symptoms often fit only generally within the modified NIDRR descriptors and may require more user friendly

For peer review only

For peer review only - http://bmjopen.bmj.com/site/about/guidelines.xhtml

language. The non-specific nature of many UTI signs and symptoms presents a challenge for the GP, who relies on the IC user's description, together with urine culture results, to determine the most appropriate course of action.[2] Cloudy or smelly urine was often cited as the first indicator and whilst some acted on this alone and visited the GP, others described how they would first attempt to self-manage and monitor for further symptoms, such as localised pain, discomfort or generalised illness. Some with co-morbidities and/or age-related problems had difficulty separating a possible UTI from other more general signs and symptoms, which means that there could be the risk of delayed diagnosis of UTI.

The language used to describe the signs and symptoms may differ from typical clinically accepted terms. This is an important finding, as such discrepancy affects the diagnosis and management of UTI amongst IC users. No participants, however, expressed concern about being unable to communicate effectively with GPs. This may be due to their on-going relationships with their GP so that language or interpretation differences may have been less relevant. Comprehension of terminology surrounding IC could be a focus for further research and help equip new IC users with the necessary confidence to advocate for themselves or to manage symptoms. The decision to seek medical assistance was influenced by (1) an individual's perception of symptom severity, (2) the level of confidence in the effectiveness of self-help strategies, and (3) the previous experience of UTI and its management by the GP.

The key role of the GP in helping the individual manage, whether with symptom-based treatment or having a repeat prescription, was seen by participants as invaluable. Although some were uncertain about the symptoms of UTI, in general, people using IC on a long term basis, become very familiar with the warning signs of health issues and want to be proactive in their care. In this study, IC users indicated management was facilitated by the positive

For peer review only - http://bmjopen.bmj.com/site/about/guidelines.xhtml

relationship and continuity with their GP who was aware of their health status and approached care on an individual basis. This interdependence of the individual and the physician is crucial and the reliance alone on laboratory testing should not define treatment in this population.[3] That self-medication with antibiotics might help IC users feel more confident and secure about managing symptoms needs further exploration.

Finally, health care professionals need to be attuned to individuals who self blame ('not hygienic enough') or lack an understanding of the aetiology of UTI. Such feelings were highlighted in at least one other study involving women (non-IC users) where they attributed UTI to 'poor hygiene', 'negligence', 'not drinking enough' or a 'penalty of growing old'.[14] In our study, some participants unnecessarily restricted their lives by following self-imposed rigid and complex procedures (i.e., not going out all day so they could catheterise only at home; restricting their diets; never using public toilets). When an IC user approaches the GP for assistance, exploring the story and probing for the effect of IC on daily life is likely to help in making a collaborative plan for evidence-based self-care. This requires the IC user to understand signs and symptoms which are relevant for them and to have the language to articulate their concerns to the GP. Many of the individuals in our study had used IC for several years and might have benefited from an updated educational session on current best practice with IC, causes of UTI, and use of antibiotics.

Strengths and limitations

This is the first qualitative study to explore views and experiences of UTI from a focussed IC user perspective. A strength is the inclusion of participants with a variety of reasons for using IC (including both neurological and non-neurological conditions). Whilst we included adults of all ages, the majority of our sample consisted of older people (mean age 66 years old), and thus, does not represent all IC users. In common with other qualitative research, the sample size was

For peer review only - http://bmjopen.bmj.com/site/about/guidelines.xhtml

small, and collecting data through interviews relies on recall of experiences. All descriptions of UTI discussed were subjective and self-reported. Diagnoses were not confirmed so that described signs and symptoms may not have been caused by UTI. Participants talked generally about their antibiotic use for UTI treatment; however, the interview schedule included no questions specifically related to attitudes to or understanding of antibiotics or the evidence related to UTI and IC. Advice on issues, such as use of urine dipsticks, may have been a misrepresentation of what the individual was actually told.

Implications for future research or clinical practice

To address the most appropriate course of action regarding UTI in IC users, there is a need to develop a more user-based UTI symptom list as well as an evidence-based algorithm for self-care and help seeking. Such a list may also assist in self-management and appropriate antibiotic use in this popoulation. Our findings showed that UTI signs and symptoms could be grouped under 5 broad categories: 1) changes to urine; 2) changes in bladder emptying; 3) fever; 4) generalised/systemic symptoms; and 5) pain/discomfort. Questions focused on these categories may be helpful for both patients and healthcare professionals in elucidating signs and symptoms of UTI. Further exploration of the subjective descriptions of UTI signs and symptoms and matching these with laboratory confirmation is needed in order to aid both the IC user and the healthcare professionals in providing best care.

CONCLUSION

The study has provided insight into how participants construct their experience of UTI and identified some opportunities for further research particularly related to on-going IC user education and follow up, antibiotic use, and self-care practices. Current guidelines[15] state that cloudy or malodorous urine in the catheterised adult (including IC users) should not be used

For peer review only - http://bmjopen.bmj.com/site/about/guidelines.xhtml

alone to differentiate asymptomatic bacteriuria from infection or as an indication for urine culture or antimicrobial therapy, yet in the current study these were commonly reported changes by IC users and were sometimes the impetus for help-seeking.

Focussed follow up according to evidence-based guidelines may assist in self-management and appropriate antibiotic use. Examining GP's perspectives would provide a balanced approach to improve our understanding of the diagnosis and management of UTI amongst IC users. This study provides the basis for developing a self-help tool which may aid identification of UTI and communication with health care professionals.

Acknowledgements

We would like to thank all the interviewees who agreed to take part and share their experiences. We thank also Samantha Sartain, PhD and Bridget Clancy, RGN, BSc for conducting part of the data collection, and Margaret Macaulay, RGN, MSc for assistance with the manuscript.

Contributors

MF was the principal investigator and was primarily responsible for the original grant application. MF, JP, MA, and SS were involved in designing the study and developing the methods. JP led the overall study team. SS collected the majority of the data. BC and MA also contributed to the data collection. IO led the analysis and interpretation of the qualitative data, and wrote drafts of the manuscripts. IO, JP, MA, KM and MF wrote drafts and critically revised the manuscripts.

Funding: This paper refers to independent research funded by the National Institute for Health Research (NIHR) under its Programme Grants for Applied Research (PGfAR) (Grant Reference Number RP-PG-0610-10078).

Disclaimer: The views expressed are those of the authors and not necessarily those of the NHS, the NIHR or the Department of Health.

Competing interests: None declared.

For peer review only - http://bmjopen.bmj.com/site/about/guidelines.xhtml

Ethical approval: Ethical permission was granted from a NHS Research Ethics Committee (NRES committee London Hampstead; REC reference 13/LO/1511; approval letter dated 2 October 2013).

Provenance and peer review: Not commissioned; externally peer reviewed.

Data sharing statement: No additional data are available.

For peer review only

References

1. Wyndaele JJ. Complications of intermittent catheterization: Their prevention and treatment. *Spinal Cord* 2002;40(10):536-41.
2. Wyndaele JJ, Brauner A, Geerlings SE, et al. Clean intermittent catheterization and urinary tract infection: Review and guide for future research. *BJU Int* 2012;110(11 Pt C):E910-7.
3. The prevention and management of urinary tract infections among people with spinal cord injuries. National institute on disability and rehabilitation research consensus statement. January 27-29, 1992. *J Am Paraplegia Soc* 1992;15(3):194-204.
4. García Leoni ME, Esclarín De Ruz A. Management of urinary tract infection in patients with spinal cord injuries. *Clin Microbiol Infect* 2003;9(8):780-85.
5. Massa LM, Hoffman JM, Cardenas DD. Validity, accuracy, and predictive value of urinary tract infection signs and symptoms in individuals with spinal cord injury on intermittent catheterization. *J Spinal Cord Med* 2009;32(5):568-73.
6. Cobussen-Boekhorst H, Hermeling E, Heesakkers J, et al. Patients' experience with intermittent catheterisation in everyday life. *J Clin Nurs* 2016;25(9-10):1253-61.
7. Kelly L, Spencer S, Barrett G. Using intermittent self-catheters: Experiences of people with neurological damage to their spinal cord. *Disabil Rehabil* 2014;36(3):220-26.
8. Shaw C, Logan K. Psychological coping with intermittent self-catheterisation (isc) in people with spinal injury: A qualitative study. *Int J Nurs Stud*;50(10):1341-50.
9. Wilde MH, Brasch J, Zhang Y. A qualitative descriptive study of self-management issues in people with long-term intermittent urinary catheters. *J Adv Nurs* 2011;67(6):1254-63.
10. Logan K, Shaw C. Intermittent self-catheterization service provision: Perspectives of people with spinal cord injury. *International Journal of Urological Nursing* 2011;5(2):73-82.
11. Jaquet A, Eiskjaer J, Steffensen K, et al. Coping with clean intermittent catherization--experiences from a patient perspective. *Scand J Caring Sci* 2009;23(4):660-6.
12. Logan K, Shaw C, Webber I, et al. Patients' experiences of learning clean intermittent self-catheterization: A qualitative study. *J Adv Nurs* 2008;62(1):32-40.
13. Fader M. Development and clinical trial of a mixed (multi/single-use) catheter management package for users of intermittent catheterisation. National Institute for Health Research (NIHR) Programme Grants for Applied Research (PGfAR) Programme (Grant Reference Number RP-PG-0610-10078) 2013.
14. Leydon GM, Turner S, Smith H, et al. Women's views about management and cause of urinary tract infection: Qualitative interview study. *BMJ* 2010;340:c279.
15. Hooton TM, Bradley SF, Cardenas DD, et al. Diagnosis, prevention, and treatment of catheter-associated urinary tract infection in adults: 2009 international clinical practice guidelines from the infectious diseases society of america. *Clin Infect Dis* 2010;50(5):625-63.

For peer review only - http://bmjopen.bmj.com/site/about/guidelines.xhtml

MULTICATH MODULE 2 SCHEDULE FOR ITERATION 3

Background questions

1. Experience of intermittent catheterisation:

    How long have you been using intermittent catheterisation?

    Do you self-catheterise? Or does somebody help you?

    How many times per day do you catheterise?

    What catheter do you use?

    Do you catheterise:

        At home only?

        When you are out?

        If yes, when you are out what kind of catheter do you use, is it the same one as for home use?

Beliefs and practices re IC routine

2. Beliefs and concerns about IC rituals, catheters, infection and sterility

    What steps do you take before you do IC?

    What is the reason you take these steps? (Probe without leading for whether or not they value sterility)

    Is your IC routine clean or sterile?

    What is sterile to you? (prompt – how is it different to clean?) What do you mean by clean?

Experience of urine infection

3. During your time using intermittent catheterisation have you ever experienced a urine infection?

    If yes, how often would you say you have a urine infection?

    Can you recall the last time you had a UTI? What happened?

4. What it feels like to have a urine infection:

    Can you describe how you feel when you think you may be developing a urine infection?

    Does anything happen beforehand when you think one is coming on?

For peer review only - http://bmjopen.bmj.com/site/about/guidelines.xhtml

For peer review only

How do you think your infection came about? Prompt (Where do you think the germs come from when you get a UTI?)

What do you do to prevent an infection?

What advice do you get/have you had from your health care professional to prevent infections?

Complete the table – UTI signs and symptoms: Where patients name a symptom or sign check with them the terminology and that the patient is happy that it sits within a category on the table. We want to understand the terminology that patients use to describe UTI symptoms.

Looking at the symptoms listed here, are these the right things that describe a urine infection from your experience? Is anything missing?

5. Seeking help:

When you feel you may have a urine infection, what do you do about it?

Prompt: antibiotics

At what point do you seek help from others?

Who do you seek help from? And what do you do?

Cleaning questions

6. Methods of cleaning:

Have you ever re-used your catheter?

If Yes, Under what circumstances? Did you clean it after its first use? How did you clean it? How did you store it between uses?

What is/would be acceptable to you?

If No, Have you ever thought about re-using your catheters?

If you were offered a method of cleaning and re-using your catheters instead of using a new one each time, what kinds of things would be important to you? [Prompts: How to clean; Where to clean; Where to store between uses]

What kind of cleaning regime is acceptable to you? [Prompts: what cleaning methods and products, what about storage storage/travelling, and lubrication?]

What would worry you about re-using your catheter? [Prompts: cleaning in a public loo, being at work, storing and transporting]

What would be good about re-using?

For peer review only - http://bmjopen.bmj.com/site/about/guidelines.xhtml

Discrete choice experiment questions

[I would now like to discuss with you the benefits and drawbacks of using disposable and reusable

Catheters]

7. What is good about your current catheter?

What do you think about single-use catheters?

What are the benefits of using a disposable or single-use catheter?

What are the drawbacks?

[in the following situations]

- At home?
- At work?
- On holiday?
- In a public place?
- Going out?
- Other?

What do you think about the idea of a re-usable catheter?

- What do you see as a benefit of re-using your catheter?
- What do you see as a drawback in re-using your catheter?

[in the following situations]

- At home?
- At work?
- On holiday?
- In a public place?
- Going out?
- Other?

- What would encourage you to use it?
- What would discourage you from using it?

Preamble: We know from previous interviews/speaking to other patients, that avoidance of infection is what matters most to everyone. We also know that other things are highly important such as:

- a comfortable insertion,
- having a catheter that is easy to use and handle,
- that comes in packaging which is easy to open,
- that your catheter is discrete,
- that you can trust your catheter/ trust in the product

As we already know all of this, we now want to explore other things around single-use catheters and re-usable catheters.

For peer review only - http://bmjopen.bmj.com/site/about/guidelines.xhtml

Together let's go through each item in this list and tell me what you think about each one? Your comments are very valuable to us.

Those relating to disposable catheters:

1. That I don't have the bother of cleaning the catheter
2. That I don't need to have special equipment for cleaning the catheter
3. That I don't have to carry used/soiled catheters around with me in my bag

Those relating to re-using catheters:

4. That I have less catheters to carry with me on holiday
5. That my catheters don't cost the NHS more than they need to/too much
6. That I don't have to store lots of catheters at home
7. That I don't have to worry that I will run out of catheters
8. That I don't have to get rid of lots of rubbish when I am out
9. That my catheters are not bad for the environment.

For peer review only

The MultICath Study (LREC: 13/LO/1511) - Urinary Tract Infection (UTI) signs and symptoms

Participant study ID: _______________ **Date of completion:** ________________ Completed by (interviewer name): ____________________

Tick all the signs and symptoms that refer to your experience of UTI:

Signs and symptoms													
	Autonomic dysreflexia	Fever	Kidney /Bladder discomfort	Increased Bladder activity (spasticity)	Feeling sick	Sense of unease	Increased catheterising	Feeling tired	Incontinence	Foul smelling urine	Cloudy urine	White blood cells in urine	Haematuria
Tick if yes													
**Circle how often you experience each symptom when you get a UTI**													
Always													
Sometimes													
Rarely													

Which of the above symptoms they mentioned would cause them to seek help from GP? __________________________

Which of the above symptoms they mentioned would cause them to take antibiotics (patients with own supply)? ____________________________

How often does the patient have a UTI? (tick which applies)

Once a week	Once a month	Every other month	Twice a year	Once a year	Less than once a year

How many times has the patient been to their GP in the last year for a UTI? ________________________________

How many times has the patient taken antibiotics in the last year for a UTI?________________________________

Have you been referred to a specialist in the last year for UTI symptoms? (eg. Urologist) _____________________

For peer review only

COREQ (COnsolidated criteria for REporting Qualitative research) Checklist

A checklist of items that should be included in reports of qualitative research. You must report the page number in your manuscript where you consider each of the items listed in this checklist. If you have not included this information, either revise your manuscript accordingly before submitting or note N/A.

Topic	Item No.	Guide Questions/Description	Reported on Page No.
**Domain 1: Research team and reflexivity**			
Personal characteristics			
Interviewer/facilitator	1	Which author/s conducted the interview or focus group?	
Credentials	2	What were the researcher's credentials? E.g. PhD, MD	
Occupation	3	What was their occupation at the time of the study?	
Gender	4	Was the researcher male or female?	
Experience and training	5	What experience or training did the researcher have?	
Relationship with participants			
Relationship established	6	Was a relationship established prior to study commencement?	
Participant knowledge of the interviewer	7	What did the participants know about the researcher? e.g. personal goals, reasons for doing the research	
Interviewer characteristics	8	What characteristics were reported about the inter viewer/facilitator? e.g. Bias, assumptions, reasons and interests in the research topic	
**Domain 2: Study design**			
Theoretical framework			
Methodological orientation and Theory	9	What methodological orientation was stated to underpin the study? e.g. grounded theory, discourse analysis, ethnography, phenomenology, content analysis	
Participant selection			
Sampling	10	How were participants selected? e.g. purposive, convenience, consecutive, snowball	
Method of approach	11	How were participants approached? e.g. face-to-face, telephone, mail, email	
Sample size	12	How many participants were in the study?	
Non-participation	13	How many people refused to participate or dropped out? Reasons?	
Setting			
Setting of data collection	14	Where was the data collected? e.g. home, clinic, workplace	
Presence of non-participants	15	Was anyone else present besides the participants and researchers?	
Description of sample	16	What are the important characteristics of the sample? e.g. demographic data, date	
Data collection			
Interview guide	17	Were questions, prompts, guides provided by the authors? Was it pilot tested?	
Repeat interviews	18	Were repeat inter views carried out? If yes, how many?	
Audio/visual recording	19	Did the research use audio or visual recording to collect the data?	
Field notes	20	Were field notes made during and/or after the inter view or focus group?	
Duration	21	What was the duration of the inter views or focus group?	
Data saturation	22	Was data saturation discussed?	
Transcripts returned	23	Were transcripts returned to participants for comment and/or	

For peer review only

Topic	Item No.	Guide Questions/Description	Reported on Page No.
		correction?	
**Domain 3: analysis and findings**			
Data analysis			
Number of data coders	24	How many data coders coded the data?	
Description of the coding tree	25	Did authors provide a description of the coding tree?	
Derivation of themes	26	Were themes identified in advance or derived from the data?	
Software	27	What software, if applicable, was used to manage the data?	
Participant checking	28	Did participants provide feedback on the findings?	
Reporting			
Quotations presented	29	Were participant quotations presented to illustrate the themes/findings? Was each quotation identified? e.g. participant number	
Data and findings consistent	30	Was there consistency between the data presented and the findings?	
Clarity of major themes	31	Were major themes clearly presented in the findings?	
Clarity of minor themes	32	Is there a description of diverse cases or discussion of minor themes?	

Developed from: Tong A, Sainsbury P, Craig J. Consolidated criteria for reporting qualitative research (COREQ): a 32-item checklist for interviews and focus groups. *International Journal for Quality in Health Care*. 2007. Volume 19, Number 6: pp. 349 – 357

Once you have completed this checklist, please save a copy and upload it as part of your submission. DO NOT include this checklist as part of the main manuscript document. It must be uploaded as a separate file.

For peer review only
